# Johann Gregor Mendel: Born to be a scientist?

**DOI:** 10.1371/journal.pbio.3001703

**Published:** 2022-07-19

**Authors:** Eva Matalova

**Affiliations:** Institute of Animal Physiology and Genetics, Academy of Sciences of the Czech Republic, Brno, Czech Republic

## Abstract

Gregor Johann Mendel, born 200 years ago, was supposed to be a farmer, intended to be a teacher and became a priest, before becoming the researcher associated with genetics we know today. This Perspective looks at his life through his own words.

## Teaser

Johann Gregor Mendel, born 200 years ago, was supposed to be a farmer, intended to be a teacher, became a priest, turned to being a researcher, and later became a world famous scientist associated with genetics. Here, we look into his life through his own words.

“His special liking for the field of natural science deepened the more he had the opportunity to become familiar with it” [1,2].

**Fig 1:**
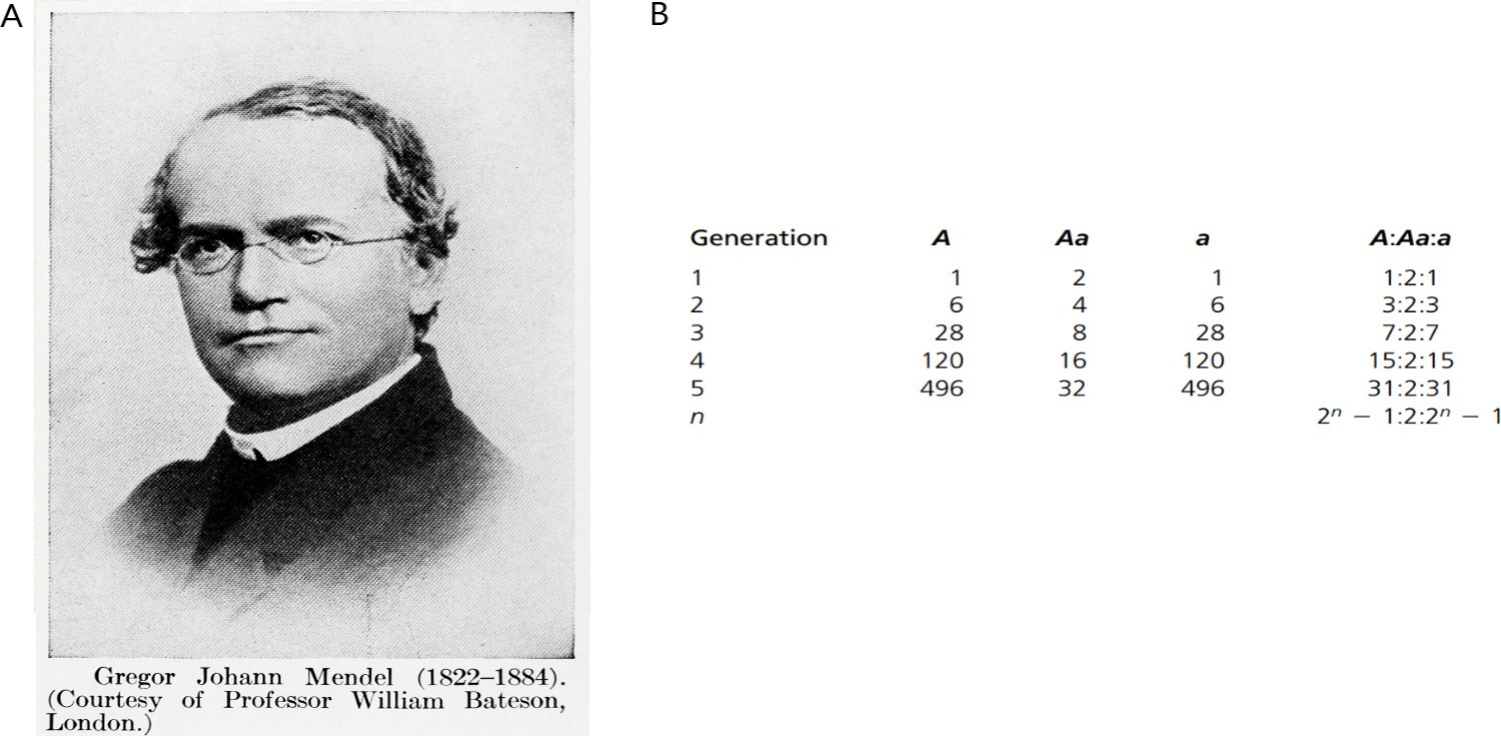
Gregor Mendel—teacher and scientist. (A) A portrait of Gregor Mendel (1822–1884). Credit: Wellcome Collection. Attribution 4.0 International (CC BY 4.0). (B) Mendel´s model expressed in terms of ratios [5,6].

Johann Mendel (born 1822) wanted to be a teacher (Fig 1A). Mendel attended a course for candidates of teaching and private teachers during his Gymnasium studies in Troppau (1834 to 1840) and, at the age of 16, received his certificate with top results and recommendations. He carried out additional studies at the Philosophical Institute in Olmütz, which he accomplished with excellent results despite setbacks owing to economic problems caused by his father’s poor health [3,4].

In his brief sketch of his life, Mendel admits that it was the miserable conditions he endured that decided his entry into the monastery in Brünn/Brno after his philosophical studies in Olmütz/Olomouc. “His circumstances decided his vocational choice” [1,2]. Thus, in 1843, 21-year-old Johann, who intended to be a teacher, turned into Gregor, a priest. Mendel started his duties but it was clear that he was unsuitable for such role. As a way out, Mendel was offered a position as a substitute teacher in Znaim/Znojmo, which he accepted with enthusiasm. To get the necessary teacher’s certification for further teaching and to “…gain the fulfilment of his wish” [1,2], Mendel applied for examinations in Vienna, Austria.

Although his 2 attempts to pass the examination were not successful, Mendel remained a dedicated (ever substitute) teacher and even received several official acknowledgments for his contribution to the balance of mathematical, natural scientific, and technical subjects with the humanities and for encouraging his pupils to pursue science. Mendel enjoyed teaching at the newly established modern Realschule in Brno, gathering several outstanding personalities. The Realschule became the seat of the Naturforschender Verein (Natural Research Society), a strongly scientifically oriented filial organization of the Agricultural Society. The declared objective of the Naturforschender Verein was to discover the laws of nature and to explain its creative forces using a material basis. At the Verein meetings, Mendel presented the results of his experiments with peas in a lecture series in 1865 that was printed in the *Society* journal the following year as the later world-famous publication Versuche über Pflanzen-Hybriden [6]. Mendel was intensely engaged in the Agricultural Society from 1851 until his death, was very active in several of its sections, and had a kind of collegium there. Communication within this Society was a source of inspiration for Mendel in his research.

Mendel dedicated all his spare time to investigation, using precisely designed experiments that he approached with scientific optimism. “As must be expected, the experiments proceed slowly. In the beginning, some patience is required, but later, when several experiments are progressing concurrently, matters are improved. Every day, from spring to fall, one’s interest is refreshed daily, and the care which must be given to one’s wards is thus amply repaid. In addition, if I should, by my experiments, succeed in hastening the solution of these problems, I should be double happy” [7,8].

His later famous paper was an outcome of years of hard and precise (intellectually as well as manually) experiments including preparation by setting defined starting conditions. “That a generally standard law for the formation and development of hybrids has not yet been successfully given is no wonder to anyone who knows the extent of the subject and who realises the difficulties with which experiments of this kind must struggle. A final determination will result only when detailed experiments on the most diverse plant families are available. Anyone who surveys the work in this area will be convinced that among the numerous experiments, none has been carried out in the extent and manner that would make it possible to determine the number of the various forms in which the progeny of hybrids appear, so that one could, with confidence, arrange these forms into the individual generations and determine their relative numerical relationships. Some courage is certainly required to undertake such an extensive work; nevertheless, it seems to be the only proper means to finally reach resolution of a question regarding the evolutionary history of organic forms, the importance of which must not be underestimated” [5,6].

“Evidently, we are here dealing only with individual phenomena that are the manifestation of a higher, more fundamental, law” [6,7]. The equilibrium of dominant and recessive elements was explained in his Experiments on Plant Hybrids and expressed in terms of ratios (Fig 1B). This is the first model in biology, calculated by Mendel the physicist [4].

“I knew that the results I obtained were not easily compatible with our contemporary scientific knowledge, and that under the circumstances publication of one such isolated experiments was doubly dangerous; dangerous for the experimenter and for the cause he presented.…I attempted to inspire some control experiments, and for that reason discussed the *Pisum* experiments at the meeting of the local society of naturalists. I encountered, as was to be expected, divided opinion; however, as far as I know, no one undertook to repeat the experiments. When, last year, I was asked to publish my lecture in the proceedings of the society, I agreed to do so, after having re-examined my records for the years of experimentation, and not having been able to find a source of error. …These and similar experiments on the germ cells appear to be important, for I believe that their results furnish the explanation for the development of hybrids as observed in *Pisum*. These experiments should be repeated and verified” [7,8].

The happy years at the Realschule and in the Agricultural Society came to an end in 1868 when Mendel was elected abbot of the monastery. “Recently, there has been a completely unexpected turn in my affairs.…From the very modest position of teacher of experimental physics, I thus find myself moved into a sphere in which much appears strange to me… This shell not prevent me from the continuation of the cross-breeding experiments… I even hope to be able to devote more time and attention to them…” [7,8]. This hope, however, was not fulfilled. Mendel was overloaded and tired out by administration and bureaucracy that was additionally increased by his position as the vice-director, and later director, of the Mortgage Bank.

He died at the age of 61. At Mendel’s death, no one officially recognized the significance of his scientific contribution except the Agricultural Society (in obituary). “His experiments opened a new epoch. What he did will never be forgotten” [9]. The world eventually acknowledged his contribution to science 16 years later. To the present day, his ideas, modern attitude, and way of scientific critical thinking made his legacy ever living [10].
